# Does Online Social Support Affect the Eating Behaviors of Polish Women with Insulin Resistance?

**DOI:** 10.3390/nu16203509

**Published:** 2024-10-16

**Authors:** Katarzyna Magdalena Pastusiak, Matylda Kręgielska-Narożna, Michalina Mróz, Agnieszka Seraszek-Jaros, Wiktoria Błażejewska, Paweł Bogdański

**Affiliations:** 1 Department of Treatment of Obesity, Metabolic Disorders and Clinical Dietetics, Poznan University of Medical Sciences, 60-355 Poznan, Poland; matkreg@ump.edu.pl (M.K.-N.); 88040@student.ump.edu.pl (W.B.);; 2 Faculty of Medical Sciences, Poznan University of Medical Sciences, 61-701 Poznan, Poland; 3 Department of Bioinformatics and Computational Biology, Poznan University of Medical Sciences, 60-812 Poznan, Poland; seraszek@ump.edu.pl

**Keywords:** insulin resistance, support groups, healthy diet, eating behaviors

## Abstract

Background: Insulin resistance, a key factor in developing type 2 diabetes mellitus, is linked to various health conditions. The basis of its treatment is lifestyle modification. However, adherence to nutritional and other medical recommendations can be challenging for chronic disease patients due to many factors, including demographics, social context, gender, age, and the patient’s baseline health condition. This study aims to evaluate the impact of online support group members on the diet quality of women with insulin resistance. Methods: This study was conducted as an online survey consisting of KomPAN (validated food frequency questionnaire) augmented with questions regarding using professional counseling and membership in support groups. The study covered 1565 women with insulin resistance, 1011 of whom were associated with the online support group. Results: The mean pHDI (pro-health diet index) was 5.18 ± 2.69 in the support groups and 4.86 ± 2.69 in the control group (*p* = 0.0319. There were no significant differences in the nHDI (non-health diet index). We found that the pHDI is associated with financial situations, the household’s situation, occupation education level, and medical or dietitian care, whereas occupation, medical, and dietitian care affect the nHDI. Membership in support groups is related to a higher pHDI and state of self-assessment of nutritional knowledge. Conclusions: Our study indicates a relationship between participation in online support groups and dietary behaviors and the subjective assessment of nutrition knowledge. Future research should focus on elucidating the mechanisms behind these influences and exploring how these communities can be optimized for broader public health initiatives.

## 1. Introduction

Insulin resistance (IR) is a metabolic condition characterized by diminished sensitivity of peripheral tissues to the biological effects of insulin, leading to impaired glucose uptake and utilization alongside augmented hepatic gluconeogenesis [1]. Insulin resistance is a critical component in the pathogenesis of type 2 diabetes mellitus (T2DM). It is associated with obesity, dyslipidemia, hypertension, polycystic ovary syndrome (PCOS), non-alcoholic fatty liver disease, and some cancers [2,3,4,5]. The etiology of IR is multifaceted, involving a complex interplay of genetic predisposition and environmental and lifestyle factors [6].

Treatment of IR primarily involves lifestyle modification, especially weight management, diet, and physical activity. Recommendations for patients with IR are consistent with recommendations for patients at high risk of type 2 diabetes [7]. The most crucial goal in those subjects is to achieve an appropriate energy balance, which leads to maintaining proper body weight. It can be achieved by adhering to various dietary patterns that align with the general principles of healthy eating [8].

Adherence to nutritional and other medical recommendations is often a very demanding task. Research indicates a low level of adherence to recommendations in chronic conditions. A notable example is the large EUROASPIRE study conducted among individuals with coronary heart disease, in which less than one-third of respondents partially adhered to recommendations for secondary prevention of heart disease [9]. Many factors influence adherence to lifestyle changes. Major determinants include demographics, social context, gender, age, and the patient’s baseline health condition [10]. Inadequate compliance leads organizations like the American Diabetes Association (ADA) or the European Association for the Study of Obesity (EASO) to emphasize the need for support for people coping with non-communicable diseases [11,12]. Actions encouraging patients to make lifestyle changes should include individual consultations and broadly understood education in various forms. Social support can be a significant precursor to self-efficacy beliefs and behavioral alterations. In accordance with social cognitive theory, individuals perceiving social support are inclined to possess stronger self-efficacy beliefs, thereby positively impacting behavior modifications, which can be helpful in therapy [13]. One of the possibilities for building social support for patients with chronic diseases is support groups, including online communities. The main aim of those support groups is to engage and empower individuals living with IR, diabetes, or obesity in a safe, supportive, and interactive way. This study aimed to evaluate if membership in an online support group influences diet quality among women with IR, which could be helpful in the development of new initiatives and health promotion channels for patients with chronic conditions, including insulin resistance, obesity, and diabetes.

## 2. Materials and Methods

### 2.1. Consent of the Bioethics Committee

This study received the approval of the Bioethics Committee of the Poznan University of Medical Science on 6 April 2021.

### 2.2. Characteristics of the Study Population

This study covered 1565 women with insulin resistance, 1011 of whom were associated with the Insulin Resistance—Healthy Diet and Healthy Life Foundation support group.

### 2.3. Inclusion Criteria

The inclusion criteria were as follows:

- Age between 18 and 65 years;
- Insulin resistance (disorder diagnosed by a physician in the past);
- Current good health.

### 2.4. Exclusion Criteria

The exclusion criteria were as follows:

- All chronic diseases and condition, including the following:
  ○ Heart;
  ○ Liver;
  ○ Kidney;
  ○ Pancreas diseases;
  ○ Autoimmune diseases;
  ○ Metabolic diseases;
  ○ Inflammatory bowel disease;
  ○ Type 2 diabetes.
  ○ Exceptions: secondary hypercholesterolemia; secondary arterial hypertension; Pregnancy and breastfeeding; Previous or ongoing cancer; Pharmacotherapy (exceptions: hormonal contraception, metformin used in the treatment of PCOS); Mental disorders, including eating disorders; Acute inflammatory disease.

### 2.5. Research Model

**Figure 1:**
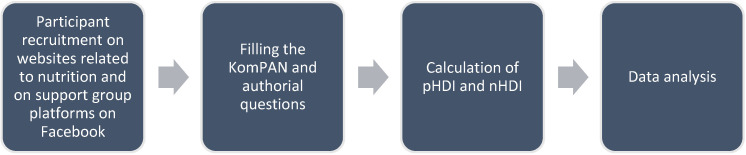
Research model.

The research model of this study is illustrated in Figure 1. This study was conducted as an online survey. Information about the study was made available online on websites related to nutrition and on support group platforms on Facebook (“Insulin resistance—diet and eating plans”—Insulin Resistance—Healthy Diet and Healthy Life Foundation support group).

The support groups serve individuals experiencing IR. Membership within these groups may be temporary, allowing for flexibility in participation. Specialists, including dietitians and medical doctors, alongside patient administrators, moderate the groups. These support groups are designed with an educational focus, providing a range of informational materials, free webinars, and question-and-answer sessions. Additionally, they offer a space for patients to share their experiences regarding lifestyle modifications, fostering a supportive community to enhance understanding and management of IR.

All participants were requested to complete the KomPAN survey, which focuses on dietary knowledge and behaviors. KomPAN is a food frequency questionnaire validated among Polish individuals aged 16 to 65. The instrument comprises four distinct sections, each containing thematically grouped questions: eating habits, frequency of food consumption, views on food and nutrition, and lifestyle and personal data. This structured approach comprehensively evaluates dietary patterns and associated lifestyle factors within the target demographic.

**Figure 2:**
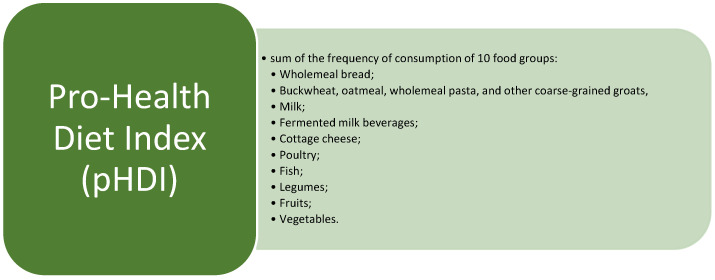
Product groups included in the calculation of pHDI.

**Figure 3:**
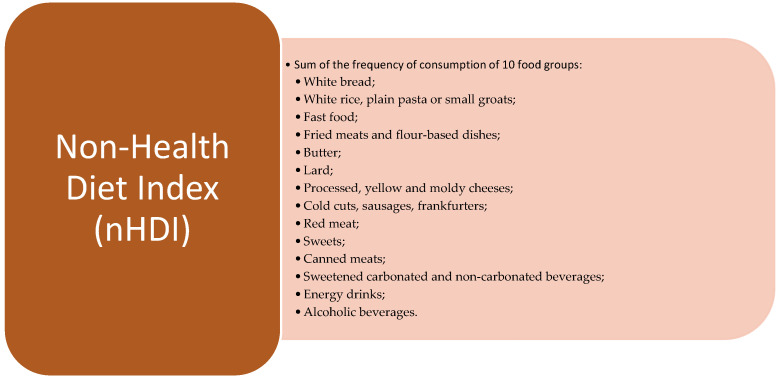
Product groups included in the calculation of nHDI.

The responses obtained from the questionnaire facilitate the calculation of both a pro-health diet index (pHDI) and a non-health diet index (nHDI). The pHDI is calculated based on the frequency of consumption of food products associated with potential health benefits (Figure 2.). The nHDI is calculated based on the frequency of food product consumption that may negatively impact health (Figure 3.). These indexes provide a quantitative assessment of dietary patterns associated with health outcomes, highlighting the consumption of foods that may contribute to health risks. The survey was augmented with inquiries regarding professional counseling and membership in support groups [14].

The assessment of the diet was carried out among all study participants, members of Insulin Resistance (study group) and non-members (control group), by completing an online form including food frequency questionnaire supplements for people aged 16 to 65 (KomPAN 2020) [14]. The data were collected between May 2021 and December 2023.

### 2.6. Statistical Analysis

The statistical analysis was conducted using Dell Statistica (data analysis software system), version 13. software (Dell Inc., 2016; Palo Alto, CA, USA). Quantitative data were presented using the mean ± SD, and the Shapiro–Wilk test was employed to verify the normality of the distribution for these variables. As most variables did not pass the normality test, the non-parametric Mann–Whitney U test was used for comparative analysis between the two groups. A comparison of more than two groups was performed using the Kruskal–Wallis test with Bonferroni’s test as a post hoc analysis. The correlation analysis between pHDI and nHDI with anthropometric parameters was performed using Spearman’s rank correlation test. Qualitative data were presented using frequency (N) and percentage (%). The analysis of relationships between qualitative variables was performed using the Chi-square test. The results were considered significant at *p* < 0.05.

## 3. Results

### 3.1. Cohort Characteristic

The primary characteristics of the study group are shown in Table 1.

### 3.2. Anthropometric Parameters

A weak negative correlation between pHDI and body mass index, body mass, and waist circumference was observed in the overall and stratified subgroups. Regarding the nHDI index, we only confirmed a weak positive correlation in the total and without a support group (Table 2).

### 3.3. Financial Situation

In the total study cohort, significant differences in the pHDI values were observed between individuals with above-average financial situations and those with below-average financial situations (*p* = 0.0088). A similar disparity was noted within the subgroup receiving support; individuals with above-average economic conditions exhibited a higher pHDI than those with below-average financial situations (*p* = 0.0163). Conversely, no significant differences were found in the group lacking support, nor concerning the nHDI index.

### 3.4. The Household’s Situation

Concerning household circumstances, a significant difference in pHDI values was identified between individuals living modestly and those living well (*p* = 0.0164) within the overall study population. In the subgroup receiving support, a similar disparity was observed. The individuals living modestly exhibited lower pHDI compared to those living well (*p* = 0.018). However, no significant differences were noted in the group lacking support, particularly concerning the nHDI index.

### 3.5. Education Level

In the subgroup receiving support, a significant contrast was observed in pHDI values between individuals with secondary education (lower pHDI) and those with higher education (*p* = 0.0092). No significant differences were noted in the overall study population or the subgroup without support, particularly concerning the nHDI index.

### 3.6. Occupation

In the overall study cohort, significant differences in pHDI were identified between individuals currently studying (higher pHDI) and retirees (*p* = 0.0130). Additionally, within the group lacking support, notable disparities were observed between individuals running the house (higher pHDI) and those working part-time (*p* = 0.038), as well as between students and part-time workers (*p* = 0.0138). No significant differences in pHDI were found in the subgroup receiving support.

Regarding nHDI, significant differences were also detected within the total group, specifically between students (higher nHDI) and retirees (*p* = 0.0322), as well as between individuals with permanent employment (*p* = 0.0182). Furthermore, disparities were noted between students (higher nHDI) and those working part-time (*p* = 0.0203) in the support group individuals. No significant divergences were observed in the group without support.

### 3.7. Medical Care

Significantly higher pHDI indexes were observed for those who had provided medical care in the group without support (*p* < 0.0071). On the other hand, nHDI index divergences were noticed, but only in the support group (*p* < 0.0006).

### 3.8. Dietitian Care

The significant differences between the pHDI indexes of people who provided dietetic care (higher pHDI) were observed overall and in subgroups (Table 3). No relation was observed when it came to nHDI (Table 4).

### 3.9. Self-Assessment of Eating Behavior

There were no significant differences in pHDI related to self-assessment eating behavior. Regarding the nHDI, significant differences were found within the total group, specifically between good (higher nHDI) and very good (*p* = 0.0332). The difference in the group without support was between the very bad (higher nHDI) and the very good states of knowledge (*p* = 0.0052).

### 3.10. Membership in Support Group

Membership in support groups (Table 5.) is related to a significantly higher pHDI index. Moreover, it was observed that there is a relationship between membership in a support group and the self-assessment state of nutritional knowledge (Chi-squered = 15.77, *p* = 0.0013). On the other hand, there was no association between online support and nHDI.

## 4. Discussion

Support groups have proven to be an effective means of increasing nutritional awareness and facilitating lifestyle modifications. Tarrant et al. highlighted in their study that these groups serve as a crucial resource for enabling lifestyle changes. The narratives within their research revealed that a strong sense of community and shared identity among group members significantly influenced how educational content was understood and integrated into daily life. By strengthening psychological connections with others facing similar challenges, participants could engage more deeply with the educational materials, improving their perceptions of social support. This collective experience of sharing personal stories made people look at their health problems differently. The sense of community made it easier for them to initiate and maintain lasting lifestyle changes [15].

The role of support groups has been well established across various health conditions, including the prevention of heart disease. Research suggests a relationship between participation in social support groups and blood pressure levels, which may positively impact cardiovascular risk. Furthermore, emotional support is crucial in managing other chronic conditions, such as insulin resistance. Additionally, improvements in participants’ quality of life were observed, partly resulting from improved health behaviors and increased support group involvement [16]. There is also substantial research highlighting the role of online support groups. These groups function as digital communities where people with similar problems offer mutual support and exchange information. Such platforms allow individuals to engage with the community without being physically present. Distance restrictions and physical barriers do not play an important role here. The benefits of online support groups include anonymity, flexible access, allowing members to seek support at any time, and the opportunity to connect with a broader range of experiences. Support groups that bring together individuals with similar health issues provide psychological support and often also serve as a source of knowledge about specific diseases [17]. The significance of support groups in managing chronic diseases is highlighted by a 2009 systematic review analyzing studies on groups of individuals with depression. Emotional support was the most commonly cited reason for participation, with many members reporting symptom improvement due to their involvement. A notable number of participants also gained valuable knowledge about medications and half of them expressed that they were able to discuss topics they felt uncomfortable addressing elsewhere. Additionally, a common benefit was a reduced sense of isolation. These groups were also found to positively impact formal help-seeking behaviors, motivating individuals to seek professional medical advice. Users further emphasized the importance of receiving support, empathy, and validation from peers, as well as having a safe space to express their feelings and find solace when feeling isolated [18]. Studies suggest that participation in support groups can provide long-term health benefits. Gallant showed that social support not only improves individuals’ self-efficacy in implementing health-related changes but also promotes lasting positive behaviors, such as maintaining regular physical activity and a balanced diet [19]. This highlights the ongoing role that social support can play beyond initial participation, reinforcing healthy lifestyle choices over time. The insulin resistance group, which was the subject of this study, serves both informational and supportive functions. It is moderated by health specialists who focus on insulin resistance, including doctors, dietitians, and physiotherapists. Educational information is published on the group’s forum, but individual opinions from group members are also shared, which may pose risks related to content manipulation and challenges for moderators. Despite some potential shortcomings of this form of communication, online forums encourage using such platforms to deepen knowledge about particular conditions. Research on forums and support groups for individuals with obesity suggests that significant factors influencing the choice of this educational medium are the lack of time healthcare professionals have to provide credible information about obesity treatment, **a** lack of trust in these professionals, the shame associated with seeking help, and a lack of connection with the physician [20]. We hypothesize that a similar situation applies to patients experiencing insulin resistance. Our study indicates that membership in the support group is positively associated with higher scores on the pro-health diet index (pHDI), as evaluated using the KOMPAN questionnaire. This is attributed not only to the dissemination of educational content related to insulin resistance within the forum and group but also to the supportive role that the group provides. This finding aligns with research by Cheung et al., which demonstrated the beneficial effects of online support group participation for individuals with obesity. The findings indicate that most participants valued closed group chats as an effective interactive platform for support; however, engagement with these chats diminished over time. Researchers emphasize that health coaches and moderators played a pivotal role in maintaining engagement and fostering community among participants [21]. In this context, the moderators’ educational and professional background is crucial. As highlighted in the analysis by Huh et al., 58% of moderator responses involved clinical expertise, with most of these provided by health professionals. Those with a health background were the main contributors of clinical knowledge across all communities, aligning with the needs of patients in these online forums [22]. The role of online weight loss communities has also been examined by Hwang et al. Their qualitative study demonstrated that these communities play a vital role in supporting participants’ weight loss efforts, offering assistance that healthcare professionals may not provide fully or close relationships in offline environments. These communities provide support comparable to in-person interactions. However, additional benefits include convenience, privacy, and lack of judgment. This type of support is considered helpful both in losing weight and in dealing with the challenges of being overweight and stigmatized [23]. A similar observation, indicating that a sense of belonging can enhance self-assessment and promote healthier dietary choices, was also reported by Reading and colleagues [24]. In our study, we likewise identified a connection between the self-perceived level of nutritional knowledge and the pro-health diet index.

Recognizing that many factors can affect the pro-health diet index is essential. Our analysis found that, beyond participation in support groups, other determinants such as financial status, household composition, education level, employment situation, and access to medical and dietary care significantly influence the pro-health diet index. These issues have been the subject of numerous studies and continue to be a pressing challenge [25,26,27]. The multitude of factors related to knowledge levels and the development of health-promoting behaviors poses a considerable challenge for researchers and practitioners alike. This complexity highlights the need to use diverse and multi-faceted health promotion interventions targeting different situations and challenges to shape and support health-promoting behaviors effectively. We acknowledge certain limitations of this study, particularly related to the multi-factorial nature of behavior determinants. Future research would benefit from expanding knowledge about patients’ motivations, psychosocial conditions, and additional environmental factors that may influence their level of knowledge and dietary behaviors. However, the large sample size is a significant strength of our research. It offers an essential contribution to the ongoing debate on how to conduct education and support individuals with chronic diseases who utilize mass media.

## 5. Conclusions

In summary, our study indicates a relationship between participation in online support groups and dietary behaviors and the subjective assessment of nutrition knowledge evaluated using the KOMPAN questionnaire. However, many additional factors may influence these behaviors and knowledge. Our findings emphasize the need to carefully examine online communities that bring together individuals with similar health conditions, particularly regarding their potential use in health promotion and prevention efforts.

This study suggests that the traditional therapeutic approach, including individual counseling and treatment, would benefit from adding social support through participation in online support groups. Future research should focus on elucidating the mechanisms behind these influences—whether they stem from the provision of resources, the fostering of personal agency, or the dissemination of reliable information—and explore how these communities can be optimized for broader public health initiatives, particularly in managing chronic conditions such as insulin resistance, obesity, and diabetes.

## Figures and Tables

**Table 1:** Characteristics of the study group.

Elements	Total = 1565 (%)	With Support Group = 1011 (%)	Without Support Group = 554 (%)	*p*
Age	34.65 ± 8.07	35.26 ± 8.03	33.53 ± 8.03	<0.0001 ^a^
Weight [kg]	81.80 ± 19.20	83.21 ± 19.23	79.22 ± 18.89	<0.0001 ^a^
Waist circumference [cm]	96.02 ± 16.01	96.80 ± 15.12	94.45 ± 17.57	0.0266 ^a^
Body mass index [kg/m^2^]	29.70 ± 8.56	30.10 ± 7.52	28.99 ± 10.15	0.0001 ^a^
Place of residence				
Village	323 (20.6)	186 (18)	137 (25)	0.0242 ^d^
City under 20,000 inhabitants	178 (11.4)	123 (12)	55 (10)	
City from 20,000 to 100,000 inhabitants	330 (21.1)	217 (21)	113 (200	
City over 100,000 inhabitants	734 (46.9)	485 (48)	249 (45)	
Financial situation				
Above average	319 (20.4)	196 (19)	123 (22)	ns ^d^
Average	1138 (72.7)	747 (74)	391 (71)	
Under average	108 (6.9)	68 (7)	40 (7)	
The household’s situation				
We live very well—we can afford some luxury	77 (4.9)	45 (4)	32 (5.5)	0.0036 ^d^
We live well—we have enough for a lot without saving much	720 (46)	451 (45)	269 (48.5)	
We live on average—we have enough for everyday life. but we have to save for more serious purchases	695 (44.4)	478 (47)	217 (39)	
We live modestly—we have to be very frugal daily	66 (4.2)	35 (3.5)	31 (6)	
We live very poor—we don’t even have enough for our basic needs	7 (0.4)	2 (0.5)	5 (1)	
Occupation				
No, I am retired or on a disability pension	17 (1.1)	12 (1)	5 (1)	0.0058 ^d^
No, I am on parental leave, I am unemployed, I run the house	163 (10.4)	122 (12)	41 (7.5)	
No, I am studying or learning	113 (7.2)	61 (6)	52 (9.5)	
Yes, but I work part-time	93 (5.9)	55 (5.5)	38 (7)	
Yes, I have permanent employment	1179 (75.3)	761 (75.5)	418 (75)	
Education level				
Higher	1169 (74.7)	765 (76)	404 (73)	0.3450 ^d^
Secondary	362 (23.1)	225 (22)	137 (25)	
Basic vocational	23 (1.5)	16 (1.5)	7 (1)	
Primary	11 (0.7)	5 (0.5)	6 (1)	
Medical care				
Yes	1332 (85.1)	874 (86)	458 (83)	0.0447 ^d^
No	233 (14.9)	137 (14)	96 (17)	
Dietitian care				
Yes	1183 (75.6)	749 (74)	434 (78.5)	ns ^d^
No	382 (24.4)	262 (26)	120 (21.5)	
Self-assessment of nutrition knowledge				
Insufficient	226 (14)	128 (13)	98 (18)	0.0013 ^d^
Sufficient	593 (38)	372 (37)	221 (40)	
Good	576 (37)	405 (40)	171 (30)	
Very good	170 (11)	106 (10)	64 (12)	
Self-assessment of eating behavior				
Very bad	55 (4)	36 (3)	19 (3.5)	ns ^d^
Bad	524 (33)	323 (32)	201 (36)	
Good	914 (58)	603 (60)	311 (56)	
Very good	72 (5)	49 (5)	23 (4.5)	
Diet Indexes				
pHDI	5.06 ± 2.68	5.18 ± 2.69	4.86 ± 2.69	0.0319 ^a^
nHDI	3.20 ± 2.17	3.15 ± 2.17	3.27 ± 2.17	ns ^a^

*p*—*p*-value; ns—not statistically significant; pHDI—pro-health diet index; nHDI—non-health diet index; ^a^—Mann–Whitney U test; ^d^—Chi-square test.

**Table 2:** Correlation between pHDI and nHDI and anthropometric parameters.

	Total		With Support Group		Without Support Group	
	R	*p*	R	*p*	R	*p*
pHDI						
Body mass [kg]	−0.10	0.0001 ^c^	−0.09	0.0040 ^c^	−0.12	0.0035 ^c^
BMI [kg/m^2^]	−0.09	0.0002 ^c^	−0.09	0.0047 ^c^	−0.12	0.0044 ^c^
Waist circumferences [cm]	−0.12	<0.0001 ^c^	−0.12	0.0014 ^c^	−0.15	0.0047 ^c^
nHDI						
Body mass [kg]	0.06	0.0285 ^c^	0.03	ns ^c^	0.11	0.0120 ^c^
BMI [kg/m^2^]	0.07	0.0088 ^c^	0.04	ns ^c^	0.12	0.0041 ^c^
Waist circumferences [cm]	0.08	0.0050 ^c^	0.06	ns ^c^	0.14	0.0056 ^c^

BMI—body mass index; R—correlation coefficient; *p*—*p*-value; ns—not statistically significant; pHDI—pro-health diet index; nHDI—non-health diet index; ^c^—Spearman’s rank correlation test.

**Table 3:** Factors affecting pro-health diet index, pHDI.

	Total = 1565 (%)	With Support Group = 1011 (%)	Without Support Group = 554 (%)
Place of residence			
Village	5.14 ± 2.72	5.33 ± 2.66	4.89 ± 2.78
City under 20,000 inhabitants	5.03 ± 2.87	5.07 ± 2.94	4.94 ± 2.72
City from 20,000 to 100,000 inhabitants	4.78 ± 2.69	4.88 ± 2.68	4.58 ± 2.71
City over 100,000 inhabitants	5.17 ± 2.6	5.28 ± 2.63	4.95 ± 2.52
*p*	ns ^b^	ns ^b^	ns ^b^
Financial situation			
Above average	5.33 ± 2.58	5.5 ± 2.62	5.08 ± 2.5
Average	5.03 ± 2.66	5.15 ± 2.66	4.82 ± 2.64
Under average	4.58 ± 3.07	4.58 ± 3.08	4.57 ± 3.09
*p*	0.011 ^b^	0.0185 ^b^	ns ^b^
The household’s situation			
Very well	5.35 ± 3.01	5.51 ± 3.08	5.14 ± 2.94
Well	5.18 ± 2.54	5.27 ± 2.56	5.04 ± 2.52
On average	5.01 ± 2.73	5.15 ± 2.72	4.7 ± 2.73
Modestly	4.15 ± 3.0	3.97 ± 3.16	4.37 ± 2.85
Very poor	3.5 ± 2.22	4.54 ± 3.96	3.08 ± 1.64
*p*	0.0092 ^b^	0.0399 ^b^	ns ^b^
Occupation			
Retired or on a disability pension	3.35 ± 2.33	3.69 ± 2.08	2.87 ± 3.03
Parental leave, unemployed, running the house	5.32 ± 2.82	5.16 ± 2.67	5.78 ± 3.2
Studying or learning	5.6 ± 2.35	5.57 ± 2.36	5.63 ± 2.37
Working part-time	4.88 ± 2.57	5.56 ± 2.39	3.91 ± 2.55
Permanent employment	5.01 ± 2.69	5.14 ± 2.74	4.78 ± 2.58
*p*	0.005 ^b^	ns ^b^	0.0016 ^b^
Education level			
Higher	5.16 ± 2.64	5.33 ± 2.67	4.85 ± 2.55
Secondary	4.86 ± 2.74	4.77 ± 2.68	5.0 ± 2.85
Basic vocational	3.73 ± 3.2	3.89 ± 3.28	3.35 ± 3.24
Primary	4.04 ± 2.53	4.29 ± 1.92	3.84 ± 3.12
*p*	ns ^b^	0.0092 ^b^	ns ^b^
Medical care			
Yes	5.04 ± 2.70	5.24 ± 2.69	5.01 ± 2.63
No	4.51 ± 2.53	4.78 ± 2.7	4.16 ± 2.62
*p*	ns ^a^	ns ^a^	0.0071 ^a^
Dietitian care			
Yes	5.58 ± 2.62	5.7 ± 2.71	5.31 ± 2.4
No	4.9 ± 2.68	4.99 ± 2.66	4.74 ± 2.7
*p*	<0.0001 ^a^	0.0004 ^a^	0.0151 ^a^
Self-assessment of nutrition knowledge			
Insufficient	5.12 ± 2.68	5.13 ± 2.94	5.1 ± 2.3
Sufficient	5.04 ± 2.64	5 ± 2.6	5.1 ± 2.7
Good	5.01 ± 2.72	4.86 ± 2.73	5.37 ± 2.67
Very good	5.25 ± 2.68	5.12 ± 2.87	5.46 ± 2.36
*p*	ns ^b^	ns ^b^	ns ^b^
Self-assessment of eating behavior			
Very bad	4.88 ± 2.56	5.33 ± 2.64	4.02 ± 2.23
Bad	5.09 ± 2.61	4.94 ± 2.59	5.32 ± 2.65
Good	5.06 ± 2.73	4.98 ± 2.81	5.23 ± 2.58
Very good	5.05 ± 2.55	4.89 ± 2.68	5.39 ± 2.25
*p*	ns ^b^	ns ^b^	ns ^b^

*p*—*p*-value; ns—not statistically significant; pHDI—pro-health diet index; ^a^—Mann–Whitney U test; ^b^—Kruskal–Wallis test.

**Table 4:** Factors affecting non-health diet index, nHDI.

	Total (Mean ± SD)	With Support Group (Mean ± SD)	Without a Support Group (Mean ± SD)
Place of residence			
Village	3.45 ± 2.72	3.45 ± 2.61	4.89 ± 2.78
City under 20,000 inhabitants	5.03 ± 2.87	3.36 ± 2.35	3.65 ± 2.04
City from 20,000 to 100,000 inhabitants	4.78 ± 2.69	3.07 ± 2.39	3.36 ± 2.78
City over 100,000 inhabitants	5.17 ± 2.6	3.02 ± 1.79	3.05 ± 1.85
*p*	ns ^b^	ns ^b^	ns ^b^
Financial situation			
Above average	3.06 ± 1.88	3.07 ± 1.85	3.04 ± 1.93
Average	3.19 ± 2.17	3.11 ± 2.12	3.35 ± 2.27
Under average	3.67 ± 2.76	3.89 ± 3.16	3.30 ± 1.9
*p*	ns ^b^	ns ^b^	ns ^b^
The household’s situation			
Very well	3.36 ± 1.77	3.54 ± 1.68	3.12 ± 1.89
Well	3.09 ± 1.96	2.99 ± 1.9	3.27 ± 2.04
On average	3.25 ± 2.26	3.26 ± 2.34	3.22 ± 2.07
Modestly	3.53 ± 3.48	3.32 ± 3.2	3.78 ± 3.81
Very poor	3.29 ± 1.33	2.26 ± 0.54	3.7 ± 1.36
*p*	ns ^b^	ns ^b^	ns ^b^
Occupation			
Retired or on a disability pension	2.2 ± 1.57	2.18 ± 1.42	2.26 ± 2.07
Parental leave, unemployed, running the house	3.44 ± 2.72	3.33 ± 2.44	3.77 ± 3.43
Studying or learning	3.9 ± 2.76	4.19 ± 3.17	3.56 ± 2.18
Working part-time	3.14 ± 2.29	3.24 ± 2.69	3.0 ± 1.56
Permanent employment	3.11 ± 1.99	3.05 ± 1.96	3.23 ± 2.06
*p*	0.0059 ^b^	0.0127 ^b^	ns ^b^
Education level			
Higher	3.16 ± 1.99	3.11 ± 1.92	3.27 ± 2.1
Secondary	3.27 ± 2.41	3.23 ± 2.43	3.33 ± 2.38
Basic vocational	2.86 ± 3.79	3.24 ± 4.43	2.01 ± 1.54
Primary	4.94 ± 5.43	6.13 ± 8.04	3.94 ± 2.14
*p*	ns ^b^	ns ^b^	ns ^b^
Medical care			
Yes	3.18 ± 2.13	3.04 ± 2.0	3.27 ± 2.15
No	3.39 ± 2.35	3.88 ± 2.92	3.29 ± 2.3
*p*	ns ^a^	0.0006 ^a^	ns ^a^
Dietitian care			
Yes	3.18 ± 2.13	3.03 ± 2.10	3.63 ± 2.66
No	3.29 ± 2.35	2.98 ± 1.89	3.54 ± 2.49
*p*	ns ^a^	ns ^a^	ns ^a^
Self-assessment of nutrition knowledge			
Insufficient	3.48 ± 2.73	2.89 ± 2.17	3.34 ± 1.89
Sufficient	3.29 ± 2.26	3.04 ± 1.94	3.55 ± 2.89
Good	3.03 ± 1.83	2.98 ± 1.90	3.81 ± 2.39
Very good	3.03 ± 1.83	3.05 ± 1.89	3.24 ± 1.94
*p*	ns ^b^	ns ^b^	ns ^b^
Self-assessment of eating behavior			
Very bad	4.54 ± 3.64	3.76 ± 2.65	2.39 ± 1.01
Bad	3.89 ± 2.51	3.06 ± 2.04	3.46 ± 2.13
Good	2.77 ± 1.6	2.91 ± 1.84	3.60 ± 2.72
Very good	2.51 ± 2.48	3.20 ± 1.83	4.76 ± 2.51
*p*	<0.0001 ^b^	ns ^b^	0.0101 ^b^

*p*—*p*-value; ns—not statistically significant; SD—standard deviation; nHDI—non-health diet index; ^a^—Mann–Whitney U test; ^b^—Kruskal–Wallis test.

**Table 5:** Association of support group membership with diet indexes.

Membership in Support Group	Mean ± SD
pHDI	
Yes	5.18 ± 2.69
No	4.86 ± 2.64
*p*	0.0319 ^a^
nHDI	
Yes	3.15 ± 2.17
No	3.27 ± 2.17
*p*	ns ^a^

*p*—*p*-value; ns—not statistically significant; SD—standard deviation; pHDI—pro-health diet index; nHDI—non-health diet index; ^a^—Mann–Whitney U test.

## Data Availability

If justified, analyzed data are available from kpastusiak@ump.edu.pl.
